# The Role of Decision Support System (DSS) in Prevention of Cardiovascular Disease: A Systematic Review and Meta-Analysis

**DOI:** 10.1371/journal.pone.0047064

**Published:** 2012-10-10

**Authors:** Raghupathy Anchala, Maria P. Pinto, Amir Shroufi, Rajiv Chowdhury, Jean Sanderson, Laura Johnson, Patricia Blanco, Dorairaj Prabhakaran, Oscar H. Franco

**Affiliations:** 1 Department of Public Health & Primary Care, University of Cambridge, Strangeways Research Laboratory, Wort’s Causeway, Cambridge, United Kingdom; 2 Public Health Foundation of India, Indian Institute of Public Health, Hyderabad, Andhra Pradesh, India; 3 Centre for Chronic Disease Control, New Delhi, India; 4 Department of Epidemiology, Erasmus MC, Rotterdam, The Netherlands; College of Pharmacy, University of Florida, United States of America

## Abstract

**Background:**

The potential role of DSS in CVD prevention remains unclear as only a few studies report on patient outcomes for cardiovascular disease.

**Methods and Results:**

A systematic review and meta-analysis of randomised controlled trials and observational studies was done using Medline, Embase, Cochrane Library, PubMed, Amed, CINAHL, Web of Science, Scopus databases; reference lists of relevant studies to 30 July 2011; and email contact with experts. The primary outcome was prevention of cardiovascular disorders (myocardial infarction, stroke, coronary heart disease, peripheral vascular disorders and heart failure) and management of hypertension owing to decision support systems, clinical decision supports systems, computerized decision support systems, clinical decision making tools and medical decision making (interventions). From 4116 references ten studies met our inclusion criteria (including 16,312 participants). Five papers reported outcomes on blood pressure management, one paper on heart failure, two papers each on stroke, and coronary heart disease. The pooled estimate for CDSS versus control group differences in SBP (mm of Hg) was - 0.99 (95% CI −3.02 to 1.04 mm of Hg; I^2^ = 0; p = 0.851).

**Conclusions:**

DSS show an insignificant benefit in the management and control of hypertension (insignificant reduction of SBP). The paucity of well-designed studies on patient related outcomes is a major hindrance that restricts interpretation for evaluating the role of DSS in secondary prevention. Future studies on DSS should (1) evaluate both physician performance and patient outcome measures (2) integrate into the routine clinical workflow with a provision for decision support at the point of care.

## Introduction

Although numerous guidelines exist for prevention of CVD, risk factor control remains sub-optimal in high-risk patients and in those with established CVD [1]. Physician adherence to guidelines for prevention of CVD in general has been less than optimal [2], [3]. Moreover, published literature has demonstrated a ‘discrepancy between intentions and practise’ in the treatment of hypertension and have highlighted the physicians’ difficulty in following the complex clinical guidelines [4].

Decision support systems defined as ‘any intervention that provides clinicians with clinical knowledge and patient specific information to augment patient care decisions’ [5] have been introduced in the developed world as tools for implementing guidelines. Numerous systematic reviews have shown that Computerised Decision Support Systems (CDSS) when used as reminders improve preventive care, enhance clinical performance, influence clinical decision making [6], [7], [8], [9], [10], [11] and significantly improve the decision quality [12], [13].

Although DSS could be efficient and low cost tools for primary care in the prevention of cardiovascular disease, only a few studies report on patient outcomes for cardiovascular disease and the potential role of DSS for the CVD prevention remains unclear [8]. Hence, we aimed to systematically search for all the available studies that report the effect of DSS on prevention of cardiovascular disease. Furthermore, we aimed to evaluate whether this effect would differ by type of cardiovascular disorder and in primary or secondary prevention.

## Methods

We conducted a systematic review and meta-analysis of studies that evaluated the role of DSS in prevention of CVD among adults.

### Search Strategy and Eligibility Criteria

Between November 2010 and 30 Jul 2011 (last date searched) we comprehensively searched the following databases: Medline (1950 to present), EMBASE (1980 to present), the Cochrane Library (1960 to present), Scopus (1996 to present), Scielo (1997 to present), Web of knowledge (1970 to present), AMED (1985 to present) and CINHAL (1981 to present).

We used combinations of text words and thesaurus terms that included secondary prevention”[All Fields] AND (Humans[Mesh] AND adult[MeSH]))) OR (primary prevention AND (Humans[Mesh] AND adult[MeSH]))) OR (“secondary prevention”[MeSH Major Topic] AND (Humans[Mesh] AND adult[MeSH]))) OR (“primary prevention”[MeSH Major Topic] AND (Humans[Mesh] AND adult[MeSH]))) OR (“prevention”[All Fields] AND (Humans[Mesh] AND adult[MeSH])) AND (Humans[Mesh] AND adult[MeSH]))) AND (((((((((((cardiovascular diseases)) OR (cardiovascular disorders)) OR (“cardiovascular diseases”[MeSH Major Topic]))) OR ((((peripheral vascular disorders)) OR (peripheral vascular diseases)) OR (“peripheral vascular diseases”[MeSH Terms]))) OR (((“heart failure”[All Fields])) OR (“heart failure”[MeSH Major Topic]))) OR ((((((coronary heart diseases)) OR (coronary arterial diseases)) OR (“coronary arterial disease”[All Fields])) OR (“coronary heart disease”[All Fields])) OR (“coronary occlusion”[MeSH Major Topic]))) OR ((((((((brain vascular event)) OR (cerebral stroke)) OR (cerebrovascular event)) OR (cerebrovascular accident)) OR (“cerebrovascular trauma”[MeSH Major Topic])) OR (“cerebrovascular disorders”[MeSH Major Topic])) OR (“stroke”[MeSH Major Topic]))) OR (((((“heart attack”[All Fields])) OR (ischemic heart disease)) OR (myocardial infarction)) OR (“myocardial ischemia”[MeSH Major Topic]))) OR (((((diastloc blood pressure)) OR (systolic blood pressure)) OR (blood pressure)) OR (“hypertension”[All Fields])) AND (Humans[Mesh] AND adult[MeSH]))) AND ((((((((((“clinical decision support systems”[All Fields] OR “clinical decision support tool”[All Fields] OR “clinical decision support tools”[All Fields])) OR (“computerized decision support systems”[All Fields] OR “computerized decision support tool”[All Fields] OR “computerized decision support tools”[All Fields])) OR (clinical decision making tools)) OR (computeri* AND decision support systems)) OR (“clinical decision support systems”[All Fields])) OR (“decision support systems”[All Fields])) OR (“decision support systems, clinical”[MeSH Major Topic]))) OR (medical decision making)).

Studies were included if they were:

1. Cross sectional, case control, cohort and randomized controlled trials (RCTs).
2. Studies conducted among adult populations (≥18 years old).
3. Studies on prevention of cardiovascular disorders (myocardial infarction, stroke, coronary heart disease, peripheral vascular disorders and heart failure) and management of hypertension due to the types of interventions (defined in point number 4 below)
4. Studies on interventions including: decision support systems, clinical decision supports systems, computerized decision support systems, clinical decision making tools and medical decision making

Articles were excluded if they were:

1. Letters, abstracts, conference proceedings, reviews and meta-analysis
2. Not conducted in humans

Two independent reviewers working in pairs (RA and OHF, AS and JS, LJ and RC) screened the titles and abstracts of the initially identified studies to determine whether they would satisfy the selection criteria. Any disagreements about selection were resolved through consensus or consultation with a third author. Full text articles were retrieved for the selected titles. Reference lists of the retrieved articles were searched for additional publications. We also contacted the authors of the retrieved papers directly for any additional and unpublished studies. The retrieved studies were assessed again by two independent authors (RA and OhF) to ensure that they satisfied the inclusion criteria.

### Data Extraction

A data collection form was designed prior to the implementation of the search strategy. This form was used by two independent reviewers to extract the relevant information from the selected studies (RA and OhF). The data collection form included questions on qualitative aspects of the studies (e.g. date of publication, design, geographic origin and setting, funding source, selection criteria, patient samplings and location of research group), participant characteristics (e.g. number of population included in the analysis, age range, mean age, gender, ethnicity, recruitment procedures, residential region, socio economic status, comorbidities and drug treatment) characteristics of the exposure/intervention evaluated (e.g. type, method used to measure) and information on the reported outcomes (e.g. measure of disease association, type of outcome, outcome assessment method, type of statistical analysis, adjustment variables).

### Statistical Analysis

A two sample t test yielded the mean difference (SE) in SBP between both the intervention (DSS) and the control groups. A fixed effects meta-analysis model was used to pool the mean difference in SBP from all the five studies. The “I squared statistic”, which quantifies the percentage of variation attributable to heterogeneity, was reported as a measure of consistency across the studies. The mean SBP difference and the 95% CIs have been reported in the pooled analysis.

### Quality Evaluation

The quality of the studies included was evaluated using a scare of maximum of 10 points (for the highest quality) ^10^ based on the following aspects of the study: (1) *Allocation to study groups* (random, 2; quasi-random, 1; selected concurrent controls, 0); (2) *Data analysis and presentation of results* (appropriate statistical analysis and clear presentation of results, 2; Inappropriate statistical analysis or unclear presentation of results, 1; inappropriate statistical analysis and unclear presentation of results, 0); (3) *Presence of baseline differences between the groups that were potentially linked to study outcomes* (no baseline differences present or appropriate statistical adjustments made for differences, 2; baseline differences present and no statistical adjustments made, 1; baseline characteristics not reported, 0); (4) *Objectivity of the outcome* (objective outcomes or subjective outcomes with blinded assessment, 2; subjective outcomes with no blinding but clearly defined assessment criteria, 1; subjective outcomes with no blinding and poorly defined, 0); (5) *Completeness of follow-up for the appropriate unit of analysis* (90%, 2;from 80% to 90%, 1; <80% or not described, 0).

## Results

### Study Selection

**Figure 1:**
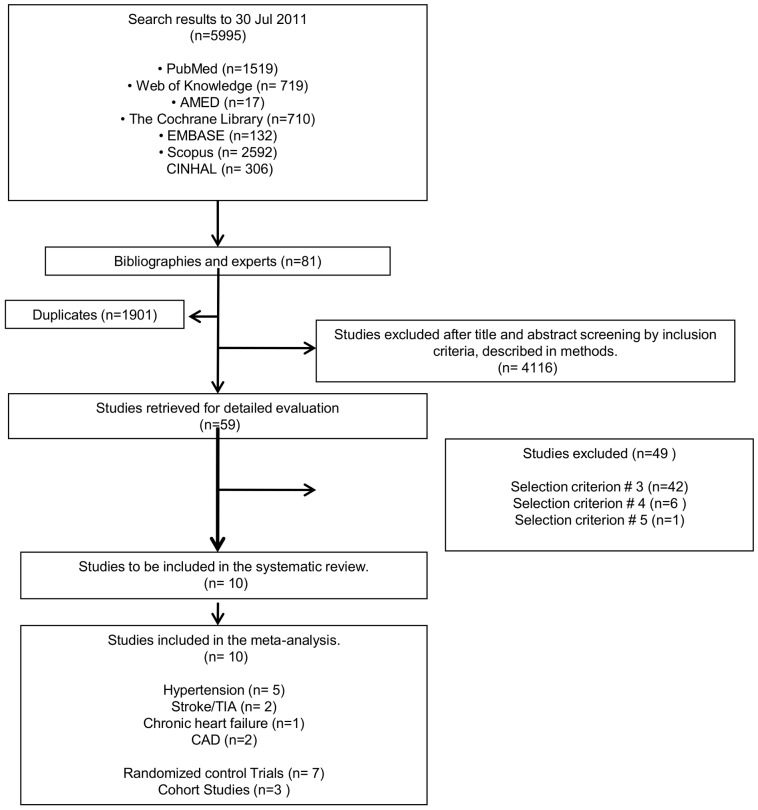
Flow diagram for the selection of studies evaluating the role of DSS in prevention of CVD.

Overall 6076 references were initially identified in our study: 5995 from electronic databases and 81 from bibliographies and experts (**Figure 1**). Full-text assessment of the 59 potentially relevant articles resulted in 10 eligible studies that were included in our analyses.

### Characteristics of Studies Included

All the papers were published after the year 2000 and came from high income and developed countries (**table 1**). Five studies were conducted in the USA [14], [15], [16], [17], [18], two in UK [19], [20], one in Israel [21], one in Australia [22], and one in Canada [23]. Five papers reported outcomes on blood pressure management [14], [15], [16], [19], [23], one on heart failure [22], two on Transient Ischemic Attack (TIA) or stroke [17], [20] and two on coronary heart disease [18], [21] (**table 1**). Seven papers were randomized trials and the remaining three [17], [18], [22], were cohort studies. Eight of the papers had a computerised decision support system (CDSS), one had a telephone linked IT supported program [23] and the remaining one [22] had written guideline reminders to physicians coupled with education to the pharmacists. All the 10 papers had the physicians as the DSS users. In addition, three papers had support systems for the nurses [21], [22], [23] and two had interventions which also involved the pharmacists [22], [23]. Three were conducted in inpatient settings [17], [20], [22]. Nine papers clearly mentioned the source for the clinical knowledge in the decision support systems (except Rinfret, 2009 [23]). Five papers reported on primary prevention [14], [15], [16], [19], [23] and the other five papers reported on secondary prevention. Two papers^18, 19^ scored poorly on the quality ratings (five on a scale of ten). The remaining eight papers were of a good quality (**Table 1**).

**Table 1:** Intervention, outcomes and effect sizes for the selected studies.

Author, year of publication	Sample size; mean age inyears (range or SD);n (%) of females	Outcome;type ofprevention	Type of intervention	Effect size	Quality rating
Mudge et al [22],(2010)	416; 75 yrs (24–100) and 78 yrs(32–102); 103 (52) and 118(54) females in the baselineand interventioncohorts respectively	Heart failure; secondary prevention	Decision support tools, reminders, education and academic detailing, and performance feedback	There was a trend to increased readmissionsattributed to heart failure: 47 (21.5%) ofintervention patientscompared to33 (16.7%)in the baseline group(OR = 1.30; 95% CI: 0.87–1.93).	8
Hayden B. Bosworthet al [14], 2009	588; 63 yrs (11); 12 (2) females	Blood pressure; primaryprevention	Computerisedclinical decisionsupport system	Estimated mean systolic BP (SE) baseline, 24Months (SE), baseline to 24 months difference (SE)andp value: (a) Reminder control:141.6 (1.4),136.8(1.6), −4.9 (1.9), .01 (b) Provider decisionsupportintervention: 139.1(1.4),136.9 (1.6), −2.1 (1.9),.27(c) Patient behavioral intervention: 138.8(1.4),136.3(1.6), −2.5(2.0), .20 (d) Combined:139.2 (1.4), 136.8 (1.7), −2.3 (2.1), .26	9
Stéphane Rinfret et al [23], 2009	223; 55 yrs (44–66) and 57 yrs(44–70); 51 (45.9) and 51(45.5) females in interventionand controlgroups respectively	Blood pressure; primaryprevention	IT supported management program	Change in the mean 24-hour ambulatory BP: consistently greater in intervention subjects forboth systolic (11.9 versus 7.1 mm Hg; p 0.001) anddiastolic BP (6.6 versus 4.5 mm Hg; p 0.007).	9
Harel Gilutz et al [21], 2009	7448 [Intervention (n = 3695)Control (n = 3753)]; 65.3 yrs (9.8)and65.9 yrs (10.2); 1375 (37.21)and 1409 (37.54) femalesin intervention andcontrol groups respectively	Coronary artery disease;secondaryprevention	Computer-based clinical decision support system	A modest yet significant decrease of event-freesurvival in the intervention arm, 57.1% vs. 59.2%(P<0.03).	8
LeRoi S Hicks et al[16], 2008	2027; median age 61, 64, 61,62 yrs; 681(65), 521 (66), 83(69)and 54 (74) females for theUsual Care (UC), Computeriseddecision support (CDS), nursepractitioners (NP) in UCand NP in CDS respectively	Blood pressure; primaryprevention	Computerized decision support	(1) Adjusted odds of BP control −0.96 (0.78–1.19)for computerised support versus usual care(2) Blood pressure controlled in n (%):Usual Care527 (45%); ComputerizedSupport 410 (48%)	9
Michael D. Brownet al [17], 2007	75; 67.1 yrs (19–100); 37 (49.3) females	TransientIschemic Attack(TIA)/stroke;secondaryprevention	Computer-based clinical support	(1) The 90-day risk ofrecurrent TIA was seven out of 75 (9.3%); 95%CI: 4.6% to 18.0%); (2) RecurrentTIA - proportion (n): 0.093 (7)(95% CI −0.05, 0.18)	5
Christianne L. Roumieet al [15], 2006	1341; 65.1 yrs (11.9), 65.5 yrs(12.0) and 64.6 yrs (12.6); 11(3.4), 15 (2.7) and 19 (4) females for Provider Education only, Provider Education and Alert, Provider Education Alert and Patient Education respectively	Blood pressure; primaryprevention	Provider education and alerts	Mean systolic blood pressure (SD), mm Hg: 157.3(11.9), 158.0 (12.4) and 156.3 (11.4) in the ‘ProviderEducation only’, ‘Provider Education and Alert’ and‘Provider Education Alert, and Patient Education’groups respectively	8
Prescription inIschaemic StrokeManagement (PRISM)Group [20], 2003	1952; median - 73 yrs (64–80) and 73 yrs (62–80); 247 (53) and 126(58) females for control andintervention groups respectivelyin phase 2	Stroke; secondary prevention	Computerised decision support system	relative risk reduction (RRR) in percentage units for ischaemic and haemorrhagic vascular events was 2.7 (−0.3 to 5.7)	8
Richard I. Levin et al[18], 2002	1628; - (35–85) yrs; 847 (52)females	Acute Myocardial Infarction; secondary prevention	Computer-based clinical decision support system (ohms|cad®)	Acute myocardial infarctions were reduced by 30%RR- 0.70 [95% CI: 0.59–0.81]	5
Alan A Montgomeryet al [19], 2000	614; 71 yrs (6), 70 yrs (6), 71 yrs(5); 123 (54), 130 (57) and 77 (4)females for the Computer supportplus chart, Chart only and Usualcare groups respectively	Blood pressure; primaryprevention	Computer based clinical decision support system	The chart only group had significantly lowersystolic blood pressure compared with the usualcaregroup (difference in means −4.6 mm Hg(95%CI: 8.4 to −0.8)	9

**Explanatory footnote**: SD – Standard deviation; UC – Usual Care; CDS – Computerised decision support system; NP - nurse practitioners; Ohms|Cad – registered name of the decision support system.

### Effect of DSS on Prevention of CVD

The heterogeneity of the study outcomes precluded the pooling of diverse outcomes that were reported. Two papers reported the effect of computer based decision support for selecting the anti-thrombotic therapy with either TIA/stroke as the primary end point [17], [20]. The selected paper on heart failure^23^ which matched our search strategy reported no difference for the composite outcome of death or readmission among the intervention and control groups. Two papers [18], [21] in which computer based clinical decision support system was used showed significant reduction in acute myocardial infarction and cardiovascular related rehospitalizations.

### Effect of DSS on Prevention of TIA/Stroke

PRISM, 2003 [20] was a cluster-randomized, controlled trial of computer-based decision support for selecting long-term anti-thrombotic therapy after acute ischaemic stroke. The median IQR % relative risk reduction (RRR) in ischaemic and haemorrhagic vascular events was 16.3 (13.1–23.8) and 16.7 (13.5–22.9) for control and intervention groups respectively. In a fairly large sample sized (n = 1952) prospective computer-based clinical support study done by Brown [17], 2007, the mean RRR attained by prescription when CDSS information was provided increased by 2.7 percentage units (95% CI −0.3 to 5.7), which was not significant. CDSS did not result in statistically significant results for the odds ratio for the optimal therapy being prescribed (OR = 1.32; 95% CI: 0.83–1.80).

### Effect of DSS on Prevention of Heart Failure

Only one paper on heart failure [22] met our selection criterion. The multi-pronged intervention (decision support tools, reminders, education and academic detailing, and regular performance feedback) improved patient care processes at the cost of higher readmission rates. There was a trend to increased readmissions attributed to heart failure: 47 (21.5%) of intervention patients compared to 33 (16.7%) in the baseline group (OR = 1.30; 95% CI: 0.87–1.93).

### Effect of DSS on Prevention of Coronary Artery Disease

In a large sample sized (n = 7448) cluster randomized trial from Israel^22^, an assessment of all cardiovascular related rehospitalizations (major and non-major cardiac effects) and all-cause mortality during the first year revealed a significant decrease of event-free survival in the intervention arm, 57.1% vs. 59.2% (P<0.03) at the end of 6 months of follow up. Levin et al [18], in a prospective cohort (n = 1628) done in USA, reported that their developed DSS (ohms|Cad) which utilised patient- specific guidelines for coronary prevention and elimination of ischemia as the knowledge base, resulted in 30% reduction in acute myocardial infarctions relative risk (RR), 0.70; 95% confidence interval (CI),0.59–0.81].

### Effect of DSS on Management of Hypertension

Four [14], [15], [16], [19] papers (which reported the effects of DSS on SBP) had reported the mean (SE) differences in SBP between baseline and end of study visit for both the intervention and control groups. The SDs were calculated as square root of the sample size in the control or intervention group times the SE. For the study which reported the p values [16] for the differences between the baseline and the repeat SBP, difference between baseline and repeat measure (for that group) was divided by the Z value (for that p value) to yield the SE. All of these were cluster randomized trials.

**Figure 2:**
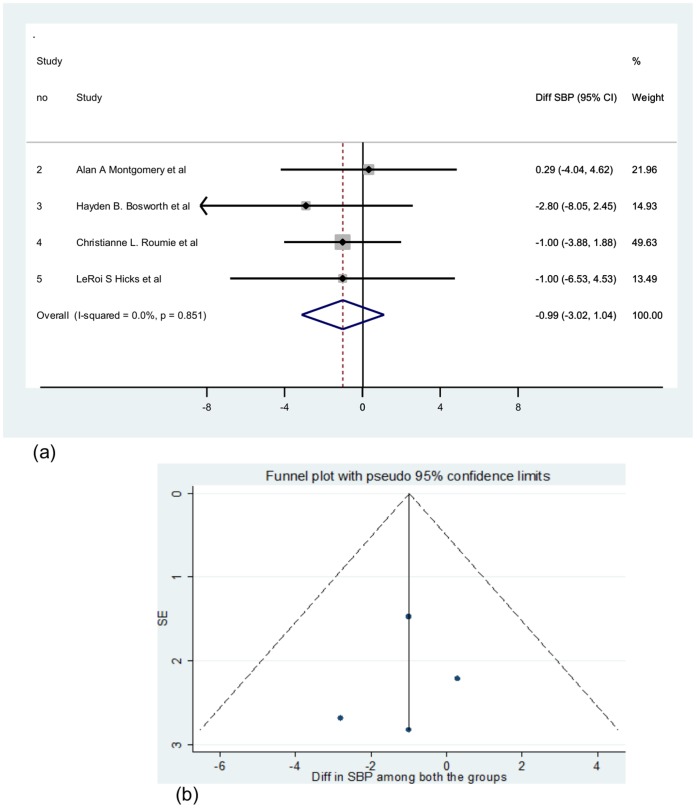
Meta analysed pooled estimate and funnel plot. 2(a): Pooled estimate for the difference in SBP (mm of Hg) between the CDSS versus control groups. 2 (b): Funnel plot to assess publication bias.

Four of the five papers on hypertension management utilised a computerised decision support system (CDSS). Figure 2(a) shows the pooled estimate for CDSS versus control group differences in SBP (mm of Hg) was - 0.99 (95% CI: −3.02 to 1.04 mm of Hg; I^2^ = 0; p = 0.851). On inclusion of the paper which reported using an Information technology assisted management program on BP control in primary care [23], the pooled estimate for the SBP difference among the intervention and control groups was −2.32 mm of Hg (95% CI: −3.96 to −0.69 mm of Hg). The I squared (variation in the estimate attributable to heterogeneity) was 27.9% (p = 0.236). Only two studies reported the DBP measure for intervention and control groups [19], [23]. The pooled estimate for DBP difference among the intervention and control group was −0.42 mm of Hg (95% CI: −2.30 to 1.47 mm of Hg) which was not significant.

To substantiate the findings of the pooled estimate a power analysis for the meta analysis on selected studies reporting effect of CDSS on SBP was done due to the small number of the studies to be included in the analysis. From the four papers reporting the mean and standard deviations of both the intervention and control group SBP (**table 2**) owing to the CDSS intervention, a pooled standard deviation (SDp), total sample size and variance was calculated. The overall mean effect size was calculated as a weighted mean with samples weighted by their variance, vi (related to their pooled sample sizes). Weights, wi, were calculated from the inverse of vi. The SDp was calculated based on the weightage given for each study.

**Table 2:** Power analysis for the meta analysis on selected studies reporting effect of CDSS on SBP.

	Study ID	n1	Mean diff SBP 1	SD 1	n2	Mean diff SBP2	SD2	Weight (wi)	weight * n	β
1	Alan A Montgomeryet al	130	0.25	1.7	202	−0.04	1.4	21.96	7290.72	0.83
2	Hayden B. Bosworthet al	143	−4.9	1.9	151	−2.1	1.9	14.93	4389.42	0.79
3	Christianne L. Roumieet al	324	−12	21	547	−11	21	49.63	43227.73	>0.99
4	LeRoi S Hicks et al	527	1	2.82	410	2	2.82	13.49	12640.13	0.88

Explanatory notes: Study ID indicates the primary authors of the study, n1 is the sample size in control group and n2 is the sample size in the intervention group, mean diff SBP1 and mean diff SBP2 are the respective mean differences in systolic blood pressures before and after the study periods, SD1 and SD2 are the respective standard deviations of both the groups, weightage is the estimated weight given to the study in the pooled analysis and β is the power of the individual studies calculated from the formula 1 mentioned in the text.

Applying the formula n  =  [2 σ2 (Z_α/2_+ Z_β_ )^∧^2/δ] – formula 1, where δ denotes the expected mean difference (or difference worth detecting), n the sample size and σ the standard deviation of the variable (pooled SD), the calculated Z_β_ using the above formula was 83.5 (when Z_α/2_ was kept at 1.96). Hence, the power of the meta analysed pooled SBP estimate was 83.5% (β = 0.83).

### Publication Bias

There was no evidence of significant publication bias after assessing the funnel plot (**Figure 2b**) for the studies that reported effects of DSS on management and control of BP.

## Discussion

CDSS did not result in significant reduction of SBP and played an insignificant role in the management and control of Blood Pressure. Although individual studies having DSS as intervention reported a decrease in the SBP measures, the pooled estimate for CDSS did not result in significant reduction in SBP (estimate of - 0.99 mm of Hg; 95% CI: −3.02 to 1.04 mm of Hg). However, when DSS was pooled with information technology assisted management program, it showed a significant pooled estimate for intervention versus control group differences in SBP (estimate of −2.32 mm of Hg; 95% CI: −3.96 to −0.69 mm of Hg). DSS moderately enhanced secondary prevention measures and slightly reduced the number of cardiovascular rehospitalizations in patients suffering from heart failure in a short time. DSS induced significant reduction in acute myocardial infarction in patients suffering from coronary artery disease. In stroke/TIA, patient related outcomes showed improvement on using the DSS or computer based clinical support.

### Comparison with the Literature

Previous systematic reviews have shown that DSS when used as reminders improve preventive care, enhance clinical performance, influence clinical decision making [6], [7], [8], [9], [10], [11] and significantly improve the decision quality [12], [13]. These reviews focused on physician performance and acceptability of the system among the health care providers. This review was attempted to study the role of DSS in primary and secondary prevention of CVDs since (1) only a few studies report on patient outcomes for cardiovascular disease and (2) the potential role of DSS in CVD prevention remains unclear.

### Lack of Focus on Preventive DSS

Traditionally, DSS interventions have focused on patient assessment and disease management at tertiary or secondary levels of care. Preventive care was left out in the bargain. In a seminal review paper on DSS, Kawamato listed the salient features that are associated with an improved physician performance and patient related outcomes. Integration of the DSS into the routine clinical workflow, maintenance of electronic templates, provision of decision support at the location of care and provision of recommendations for care (and not just assessments)^7^ are important features for a tailor made DSS.

### Strengths and Limitations of the Review

The pooled estimate for CDSS versus control group differences in SBP derived in this paper is a fair result as all the studies that reported the effect of CDSS on management and control of BP were cluster randomized trials, wherein the clinical knowledge base was based on standardized guidelines for management of BP. Further, the power of the meta analysed pooled SBP estimate was estimated as 83.5% (β = 0.83), which supports the validity of the pooled estimate for SBP. Although, the clinical knowledge base for the CDSS was different in all the studies that reported outcomes on BP control, they were tailor made for that country or condition (ATHENA hypertension guidelines decision support system [14]; Israeli guidelines for the management of dyslipidemia [21]; JNC VI and VII [16], AHA/ACC 2001 guidelines for cardiovascular disease prevention; JNC VII [15]; and New Zealand guidelines for management of hypertension [19]).

An exhaustive search of all available databases and reporting of the PRISMA checklist give our review an objective framework, upon which the conclusions have been drawn. However, integration into workflow, attitude of providers, interface design, hardware (all matter at least as much as and probably more than the content and intent of the instrument - DSS) have not been captured in our review as our focus was on ‘the effect of DSS on patient related outcomes’. Potential confounding may arise as the review had excluded studies that did not report outcomes (studies that reported benefit of DSS on medicine prescription rate, physician adherence and performance have not been included).

The heterogeneity in the included papers i.e, veteran population not representative of the general population, absence of clear delineation of effects of behavioral interventions and CDSS intervention in the management of BP [14], [23]; reliance on medical charts to measure quality of care and blood pressure control [16]; provision of only risk stratification in CDSS with no inputs on drug dosages and treatment recommendations [19]; patient loss to follow up of more than 25% [15] limit study’s findings on the effect of CDSS in the management of BP. Out of the 4 cluster randomized trials that reported the effects of CDSS on control of BP, the sample size calculations were not adjusted for intra cluster correlation in two papers [15], [16] which limits the power needed for sample size estimations.

The paucity of data available is a major hindrance that restricts interpretation for evaluating the role of DSS in secondary prevention. Absence of holistic management of all the risk factors and the change in the incidence of CAD owing to the time gap between the baselines and intervention years [18], and a very short follow up of 6 months [21] limit the findings in the role of DSS in preventing CAD. Absence of pre and post intervention measures, convenient sampling [17] and effect of the already existing local prescription guidelines (other than the CDSS) on prescribing practices [20] may limit findings on the role of DSS in prevention of stroke/TIA.

### Conclusions

DSS show an insignificant benefit in the management and control of hypertension (insignificant reduction of SBP). The paucity of well-designed studies on patient related outcomes is a major hindrance that restricts interpretation for evaluating the role of DSS in secondary prevention. Future studies on DSS should (1) evaluate both physician performance and patient outcome measures (2) integrate into the routine clinical workflow with a provision for decision support at the point of care. The coming decade will see most countries (including developing) implementing information technology for preventive health care provision and for an uniform standardization of the quality of health care given. Hence, there exists an urgent unmet need for low cost preventive DSS tools to be developed, pilot tested, implemented and evaluated to assess the role of DSS tool in preventing the rising burden of CVD.
